# Transcription profiling reveals stage- and function-dependent expression patterns in the filarial nematode *Brugia malayi*

**DOI:** 10.1186/1471-2164-13-184

**Published:** 2012-05-14

**Authors:** Ben-Wen Li, Zhengyuan Wang, Amy C Rush, Makedonka Mitreva, Gary J Weil

**Affiliations:** 1 Infectious Diseases Division, Department of Internal Medicine, Washington University School of Medicine, St. Louis, MO, 63110, USA; 2 The Genome Institute, Washington University School of Medicine, St. Louis, MO, 63110, USA; 3 Department of Genetics, Washington University School of Medicine, St. Louis, MO, 63110, USA; 4 Washington University School of Medicine, Campus Box 8051, 660 S. Euclid, St. Louis, MO, 63110, USA

**Keywords:** Nematode, Transcription, Lifecycle, Filariasis, Parasite, *Brugia malayi*

## Abstract

**Background:**

*Brugia malayi* is a nematode parasite that causes lymphatic filariasis, a disfiguring and disabiling tropical disease. Although a first draft genome sequence was released in 2007, very little is understood about transcription programs that govern developmental changes required for the parasite’s development and survival in its mammalian and insect hosts.

**Results:**

We used a microarray with probes that represent some 85% of predicted genes to generate gene expression profiles for seven parasite life cycle stages/sexes. Approximately 41% of transcripts with detectable expression signals were differentially expressed across lifecycle stages. Twenty-six percent of transcripts were exclusively expressed in a single parasite stage, and 27% were expressed in all stages studied. K-means clustering of differentially expressed transcripts revealed five major transcription patterns that were associated with parasite lifecycle stages or gender. Examination of known stage-associated transcripts validated these data sets and suggested that newly identified stage or gender-associated transcripts may exercise biological functions in development and reproduction. The results also indicate that genes with similar transcription patterns were often involved in similar functions or cellular processes. For example, nuclear receptor family gene transcripts were upregulated in gene expression pattern four (female-enriched) while protein kinase gene family transcripts were upregulated in expression pattern five (male-enriched). We also used pair-wise comparisons to identify transcriptional changes between life cycle stages and sexes.

**Conclusions:**

Analysis of gene expression patterns of lifecycle in *B. malayi* has provided novel insights into the biology of filarial parasites. Proteins encoded by stage-associated and/or stage-specific transcripts are likely to be critically important for key parasite functions such as establishment and maintenance of infection, development, reproduction, and survival in the host. Some of these may be useful targets for vaccines or new drug treatments for filariasis.

## Background

The thread-like parasitic nematodes *Wuchereria bancrafti* and *Brugia malayi* cause lymphatic filariasis (LF) in humans. LF is a deforming and disabling neglected tropical disease. An estimated 120 million people in 81 countries are infected currently, and 1.34 billion are at risk of acquiring the infection [1]. Current treatments rely on a limited number of drugs, namely diethycarbamazine, albendazole and ivermectin. Although these treatments are partially effective, the molecular effects of these drugs on filarial nematodes are not completely understood [2]. In addition, these drugs are not effective against all parasite stages. Thus, treatments often must be provided annually for the full lifespan of adult parasites, which is estimated to be 7 to 10 years. In addition, recent reports suggest that the parasites may be developing resistance to albendazole and ivermectin treatment [3]. Therefore, the search for new drug targets and effective vaccine candidates is an important priority. Improved understanding of the molecular processes responsible for parasite development and survival could help to identify new drug targets for treatment of filarial infections.

Filarial parasites have complex life cycles with five stages and 4 cuticular molts (Additional file 1 : Figure S1) [4]. Early larval development takes place in arthropod hosts, and further development and sexual reproduction takes place in vertebrate hosts. In the case of parasites that cause LF, infective larvae (L3) are transmitted to humans by mosquitoes. While L3 are developmentally arrested in mosquitoes, they resume development after entering a suitable vertebrate host, and this leads to a molt to the fourth larval larval stage (L4) after approximately 10 days. L4 undergo dramatic growth over a period of several weeks and then molt to become dioecious L5 parasites. These immature adult worms develop further over a period of approximately 6 weeks and become sexually mature adult worms. After mating, adult female worms produce thousands of motile microfilariae (Mf) that circulate in the bloodstream of infected hosts. When Mf are ingested by a susceptible mosquito species, they migrate to the thorax and develop through two molts over approximately 14 days to become L3, completing the life cycle. While changes in morphology across the life cycle of filarial worms are well documented [4], relatively little is known about the biochemical pathways and molecular processes that accompany these developmental changes. Since many of these pathways and processes must be essential for the survival of these parasites, further research in this area may lead to identification of new targets for rational design of vaccines and drugs.

Recent advances in technology have provided exciting new opportunities for research on parasites. In the case of filariasis, assembly and annotation of the genome of *B. malayi*[5], studies of differential gene expression in different parasite types (e.g., male vs. female, or after exposure to stresses such as in vitro culture, irradiation, or drugs) [6-9] and recent proteomics studies [10-13] have all contributed to a much deeper understanding of filarial biology. While generally informative, many of these studies were limited by insufficient surveying of different life cycle stages. The combined use of genomics, bioinformatics, and proteomics across the life cycle of *B. malayi* should improve our understanding of the molecular biology of molting, invasion and survival in the mammalian host, sexual differentiation, reproduction, and behavior [14,15].

In the current study, we have provided detailed information on global transcriptional profiles for seven *B. malayi* lifecycle stages: infective L3 directly from vector mosquitoes, and several stages collected from a mammalian host (Mf, L4, immature male and female worms (6 week post infection female (6WF) and male (6WM) and mature male (AM) and female worms (AF). We described transcriptional profiles associated with the dioecious and digenetic stages, and identified various sets of stage and gender-specific transcripts and transcripts that are fairly constantly expressed in all stages tested. In addition, we have related these results to recently reported data on *B. malayi* proteomes and to available expression data for *Caenorhabditis elegans*.

While our study was under review, Choi et al. [16] reported transcriptomic expression changes across the *B. malayi* lifecycle using deep sequencing. The transcriptional profiles and expression dynamics revealed by these two different approaches are similar in many ways (e.g., gender-associated expression and stage-specific expression). While detailed comparisons of results from the two studies are beyond the scope of this paper, a preliminary comparison shows that both technologies were useful for characterizing transcriptomes associated with development of *B. malayi*. One unique aspect of our study, however, was the inclusion of sexually immature adult worms (6 week post infection females and males) which allowed us to investigate molecules essential for sexual development. A comprehensive comparative bioinformatics analysis allowed us to identify many *B. malayi* homologues of *C. elegans* genes essential for reproduction or developmental arrest. This information will enhance the functional annotation of filarial genomes, and it could lead to the development of novel ways of treating or controlling filariasis based on blocking biological processes that are essential for parasite development and/or survival.

## Results and discussion

### *Overview of transcription profiles across the B. malayi lifecycle stages*

Genes with hybridization signals ≥ 2 times the mean intensity of the negative control elements in at least 3 of the 4 hybridizations were considered to be expressed. By this criterion, 11,032 transcripts were expressed in at least one stage/gender (Additional file 2), and this corresponds to ~ 60% of the probes represented on the microarray. Because the array includes probes from other related filarial species (*W. bancrofti*, 872 probes, *O. volvulus* 1,016 and *Wolbachia* 804), we compared hybridization results for different probe sets. As expected, the percentage of expressed transcripts was higher for *B. malayi* transcripts (~64%) than for *W. bancrofti* (~61%), *O. volvulus* (~56%) or *Wolbachia* (~9%) transcripts. We did not look at the stage-specific expression of *Wolbachia* genes, because poly-A labeled probes do not efficiently hybridize *Wolbachia* RNA which does not contain poly-A. We next compared results obtained with cDNA prepared from different parasite stages or sexes with those obtained with a reference cDNA sample made from pooled mRNA representing all of the life cycle stages studied to measure the relative abundance of transcripts in each parasite stage [17]. The relative abundance of transcripts over time was used to assess transcriptional profiles for each gene.

**Figure 1:**
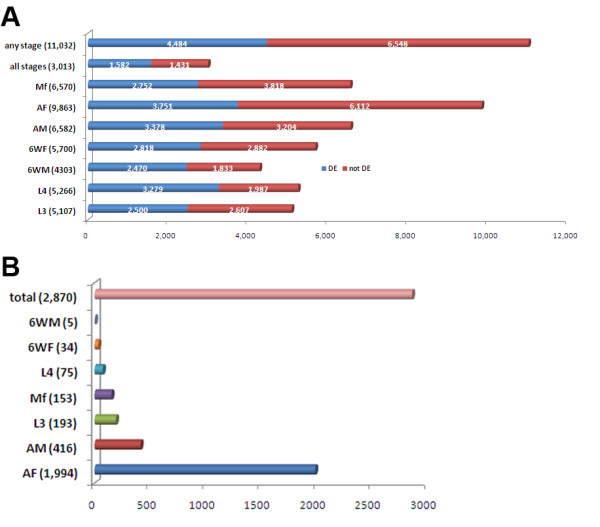
**A: This figure shows the number of the transcripts differentially (DE-shown in red) or not differentially (not DE, shown in blue) expressed in the major life cycle stages studied**. The numbers in parentheses represent the numbers of the transcripts expressed in different parasite stages. Any stage-transcripts were expressed in at least one stage. All stage- transcripts were expressed in all stages; Mf, microfilariae; AF, adult females; AM, adult males; 6WF, 6 week females; 6WM, 6 week males; L4, L4 stage, 2 weeks post infection; L3, infective third stage larvae. **B:** This figure shows the number the transcripts that were specific for each parasite stage. The numbers in the parentheses represent transcripts specific to that stage. The total indicates the sum of all stage-specific transcripts; Other abbreviations are the same as in panel A.

Regulation of expressed transcripts was highly dynamic across the life cycle. Among the expressed transcripts, 41% (4,484/11,032) of the expressed transcripts were differentially expressed (DE) across the lifecycle stages. Detailed transcriptional profiles by stage are presented in Figure 1.

Of 11,032 expressed transcripts, only (27%) 3,013 were present in all life cycle stages; these transcripts were considered to be a core set of transcripts required for all stages of the parasites. Of 3,013 core transcripts, 1,582 were differentially expressed (adj *P* < 0.05), and 1,431 were constantly expressed in all of the lifecycle stages tested (Figure 1A). The constantly expressed transcripts include those previously validated and used as endogenous controls for qRT-PCR assays such as gene BMC11678 (Bm1_09760, which encodes U2 auxiliary factor 65 kDa subunit) and gene BMC02280 (which encodes NADH dehydrogenase subunit 1) [8]. Thus, despite major morphological differences between developmental stages, different life stages share many molecular commonalities. Recent proteomics studies have shown the percentage of proteins in core proteomes varies among organisms; 15% in *B. malayi*[13], 20.7% in yeast and 7.6% in humans, respectively [18].

To our surprise, only 26% (2,870/11,032) of transcripts (including some that did (724) and others that did not meet statistical criteria for DE (2146) were expressed exclusively in one of the seven lifecycle stages studied (Figure 1B and Additional file 2). The apparent stage-specific expression of genes is not absolute, because we cannot rule out low-level expression below the sensitivity threshold of the methods we employed.

Adult females (AF) which contain eggs and developing embryos expressed more specific transcripts than other stages (1,994/9,863, Figure 1B). Our data sets for stage-specific transcripts corroborate numerous reports of stage-specific expression of individual genes in *B. malayi* including serpin in Mf (Bm1_28525) [19] and chromadorea ALT protein (Bm1_14360) in L3 [20]. The current study also identified previously unrecognized stage-specific genes that may be crucial for parasite development. For example, two female-specific transcripts encode 3′ to 5′ exonucleases (Bm1_09560 and Bm1_56820) that function as ‘proofreaders’ to edit mismatched nucleotides at the primer terminus. The transcript for mitosis-specific chromosome segregation protein (Bm1_12960) (which is essential for cell division) was also female-specific. As these genes are crucial for protein synthesis and mitosis, they are likely to be essential for female reproduction. Although the expected functions of the known genes with stage specific expression identified in this study often have clear links to development, many of the stage-specific transcripts identified encode unknown or hypothetic proteins (46.3%).

The general transcriptional profile of *B. malayi* confirms the previous hypothesis that genes which control continuous ‘house-keeping’ processes and maintain survival are constitutively expressed, while genes that have specific functions in one or more stages of development have developmentally regulated expression [21]. Based on these results, it is clear that *B. malayi* parasite development requires complex controls for gene expression.

### *Development- and function-dependent transcription patterns in B. malayi*

**Figure 2:**
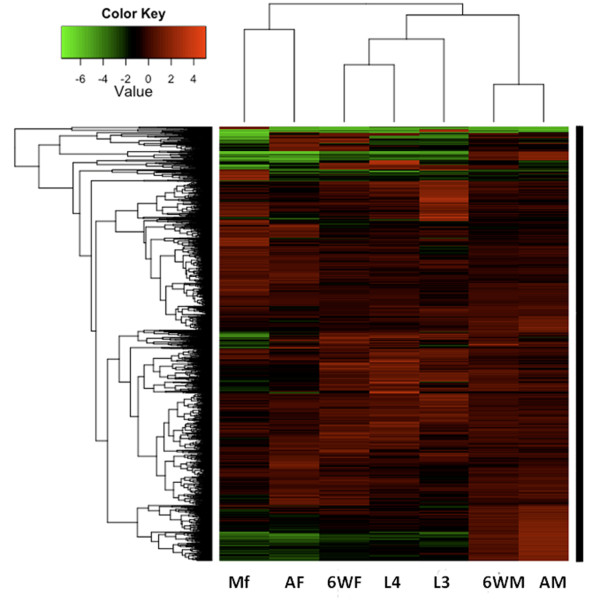
**This figure shows a heat map of 4,484 differentially expressed genes across the*Brugia malayi*life cycle stages (adj*P*< 0.05).** Mf, microfilariae; AF, adult females; AM, adult males; 6WF, 6 week females; 6WM, 6 week males; L4, L4 stage, 2 weeks post infection; L3, infective third stage larvae.

**Figure 3:**
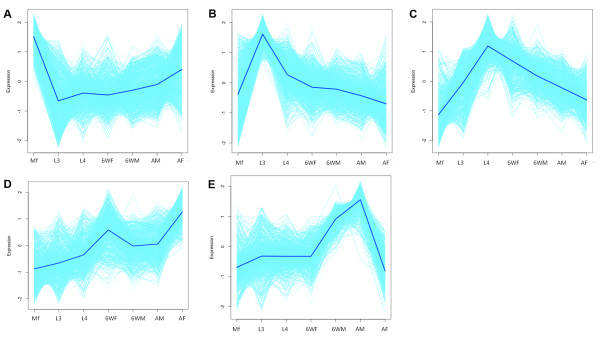
**Temporal expression patterns of 4,484*Brugia*transcripts that are differentially expressed (FDR 5%) observed over the entire life cycle**. Five clusters were identified by K-means clustering based on their temporal expression patterns. The average expression pattern of the transcripts in each cluster is indicated by a dark blue line. For visualization purposes, each transcript estimated mean log-scale expression profile was standardized to have a mean of 0 and a variance of 2 prior to plotting. **A**, Cluster one; **B**, Cluster two; **C**, Cluster three; **D**, Cluster four; **E**, Cluster five. Mf, microfilariae; AF, adult females; AM, adult males; 6WF, 6 week females; 6WM, 6 week males; L4, L4 stage, 2 week post infection; L3, infective third stage larvae.

In order to identify *B. malayi* genes that are developmentally regulated and to document their expression profiles, a statistical analysis was applied as described in Materials and Methods. We identified 4,484 probe sets (Additional file 3) that were differentially expressed with a false discovery rate (FDR) of 5% (adj *P* <0.05) when considered across the entire life cycle of *B. malayi.* This group of probe sets comprises 41% of all expressed probe sets on the microarray and represents genes that had significantly altered gene expression across the lifecycle (Figure 2). We then grouped these transcripts into five major clusters based on their temporal expression patterns using K-means clustering (Figure 3 A-E, Additional file 4). Brief descriptions of these clusters are provided in following sections.

Cluster 1 includes 1,038 transcripts with high expression in Mf. 41% of these transcripts encode hypothetical or novel proteins (Figure 3A and Additional file 4). The cluster includes many known Mf specific or enriched-genes such as serpin [19] and endochitinase [22]. One striking finding was that transcripts encoding proteins required for protein synthesis (e.g., various ribosomal protein subunits with 122 probes in the microarray, eukaryotic translation initiation factor 4E type 3 with 5 probes, and putative C_2_H_2_ domain-containing zinc finger proteins with 69 probes) were enriched in this Cluster. The C_2_H_2_ domain-containing zinc finger proteins have also been reported to be enriched in the Mf proteome [13]. These proteins may function as transcription factors. Eukaryotic translation initiation factor 4E type 3 also binds the 7-methylguanosine-containing mRNA cap during an early step in the initiation of protein synthesis and facilitates ribosome binding by inducing the unwinding of mRNA secondary structures [23]. Enrichment of transcripts for protein synthesis in Mf is consistent with the recent report of high numbers of total proteins and secretory/excretory proteins (ES) identified in Mf compared to other stages [13]. Some of these proteins may be needed for Mf survival in mammalian host and/or for infection of mosquitoes.

Cluster 2 contains 882 transcripts, 46% of which encode hypothetical or novel proteins (Figure 3B and Additional file 4). These transcripts had peak expression in the L3 stage and lower expression in other stages. Cluster 2 transcripts include most known L3-specific and/or enriched transcripts such as transcripts encoding abundant larval protein family members (BMC12455, Bm1_26880 and Bm1_14360) and transcripts encoding members of cathepsin L-like cysteine proteinase family group Ia (Bm1_00065, Bm1_00095, Bm1_00870, Bm1_13265, Bm1_20385, Bm1_53625). These well known L3 enriched proteins have been postulated to play crucial roles for establishment of infection and survival in mammalian hosts [24-27]. In addition, 54 of the 100 most highly upregulated transcripts in this cluster were exclusively expressed in L3; 14 of these encode for cathepsin L-like cysteine proteinases and cystatin ( Additional file 4).

Cluster 3 contains 786 transcripts with L4-upregulated expression (Figure 3C and Additional file 4). These transcripts exhibited peak expression in L4 with gradually reduced expression in later stages. Approximately 41% of the Cluster 3 transcripts encode hypothetical or novel proteins. Transcripts that encode proteins likely to be essential for parasite development and adaptation to stress in the mammalian host are abundant in the cluster. For example, transcripts encoding heat shock proteins (HSP) including small heat shock proteins (sHSP) BmHSP12.6 (AA389879), BmHSP70 (Bm1_43675) and BmHSP 90 (Bm1_51495) were highly upregulated in cluster 3. Previous studies have shown that sHSP are expressed in a developmentally regulated fashion [28,29]and are essential for intracellular protein folding to mediate protective functions under stress conditions [30]. Recently, BmHSP12.6 has been reported to function as a human IL-10 receptor binding protein *in vitro*[31]. In addition, transcripts encoding other stress-inducible and anti-oxidant proteins were abundant in this cluster such as the stress-inducible thioredoxin peroxidases (BmTRX, BMC12241 and AI723683), glutathione peroxidases (BmGPX, AI083432 and AA570858), and anti-oxidant products superoxide dismutase (BmSOD, BMC00677 and TC8075). These proteins have also been reported to be enriched in the secretory proteome of *B. malayi* L4 [12]. Another group of genes enriched in Cluster 3 are transcripts for cytoskeletal filaments including microfilaments (actin filaments) and intermediate filaments. This is consistent with the recent report that intermediate filament protein was abundant in ES products released during the L3/L4 transition [13]. Enrichment of transcripts for stress-inducible, anti-oxidant, and cytoskeletal proteins in this cluster suggests that L4 parasites (2 wk post infection) modulate their transcriptional program for survival and growth in the mammalian host.

Clustering analysis also revealed large groups of coordinately expressed genes with peak expression in AF (Cluster 4 with 792 transcripts, Figure 3D and Additional file 4) or in AM (Cluster 5 with 985 transcripts, Figure 3E and Additional file 4). These transcripts have high expression in 6WF or 6WM, and even higher expression in AF and AM, respectively. The percentages of the transcripts encoding hypothetical proteins or unknown proteins were higher in these clusters than that in other clusters (53% in Cluster four and 60% in Cluster five). This suggests that many transcripts associated with filarial reproduction encode novel proteins without homologues in public databases. Those proteins without close orthologs in humans may be potential targets for drugs that could block parasite reproduction. As expected, these two gender-associated clusters contained transcripts that encode known proteins involved in basic reproductive processes. For example, Cluster four (female-enriched) contains female-associated genes such as caveolin (Bm1_36280), microfilarial sheath protein BmSHP1 (Bm1_05185), and embryonic fatty acid-binding protein BmFABP (BMC00903). In contrast, Cluster five contains prominent male-associated genes such as those that that encode major sperm protein (Bm1_55755) and PDZ domain containing protein (Bm1_14680) [6,8].

We previously reported that gender-associated transcripts are more likely than other transcripts to have potential *C. elegans* homologues that are annotated as “oogenesis- or spermatogenesis-enriched” in global expression profiles [8]. This is also true for genes in Cluster 4 and 5. Cluster 4 and 5 contain many more potential homologues of *C. elegans* genes that were linked to either oogenesis or spermetogenesis, respectively, as defined by Reinke et al. [32] than other clusters (Additional file 5). This finding supports prior work that that biological functions of genes involved in core reproductive processes are highly conserved among the nematodes [33].

To demonstrate that genes with similar functions have similar expression patterns, we have investigated expression patterns of gene families identified in the *B. malayi* genome such as genes encoding kinase, nuclear receptors and genes involved in immune modulation [5]. This analysis showed that the transcriptional patterns of gene families are stage-associated and that genes with similar functions were co-expressed within clusters. For example, genes that encode proteins involved in immune modulation that may protect from host attack had Cluster 2 expression patterns with peak expression in L3 parasites; genes in the nuclear receptor family were abundant in female-associated Cluster four; genes in kinase gene family were overrepresented in the male-associated Cluster five (Additional file 6).

The expression patterns of gene families identified in this study were consistent with previous findings, and the temporal expression patterns were consistent with their likely functions in development. For example, genes encoding protein kinases are known to be highly expressed in male nematodes [8,34,35]. Prior studies have shown that kinase containing enzymes are important for regulating sperm maturation by post-translational modification of proteins [35] and in signaling cascades in oocytes following fertilization [36,37]. Likewise, high expression of the nuclear receptor gene family in female worms is consistent with prior reports [6,8] and with a recent report that 17 out of 21 nuclear receptors identified in the insect *T. castaneum* play key roles in female reproduction (especially embryogenesis) [38]. A unique property of nuclear receptors that differentiates them from other receptor classes is their ability to directly interact with and control the expression of genomic DNA. Given their important regulatory role in various biological processes, nuclear receptors have long been considered to be potential drug targets [39].

### *Stage-associated transcriptomes identified by pairwise comparisons*

The rationale behind this type of analysis is that transcriptional changes identified in pairwise comparisons may be essential for development and transition from one stage to the next. Different parasite stages encounter different environments [40,41]. This requires physical and physiological changes that are accompanied by altered expression of suites of genes with functions in many biological processes such as reproduction, development, and migration in the host [7,34,42]. Understanding these processes might help to identify new drug and vaccine targets in parasitic nematodes [43]. We performed five pairwise comparisons to identify transcripts essential for development and stage transition (L3 vs. L4, L4 vs. 6WF, L4 vs. 6WM, 6WF vs. AF and 6WM vs. AM), two pairwise comparisons to identify gender-associated transcripts (6WM vs. 6WF and AM vs. AF), and one pairwise comparison (Mf. vs. L3) to identify specific transcripts attributed to unique features of Mf and L3 stages.

Pair-wise comparisons focused on transcripts with significantly different expression (≥ 2-fold plus a FDR ≤ 0.05). The percentages of differentially expressed transcripts identified in pairwise comparisons ranged from 33% (6WM/AM) to 77% (Mf/L3). We tabulated the numbers of transcripts differentially expressed for each pairwise comparison and the numbers of upregulated transcripts in the developmental stages that comprise each pair (Table 1). The transcripts identified in these comparisons are provided in Additional file 7. Results from the pairwise comparisons are described in further detail in the following sections.

**Table 1:** Differentially expressed (DE) transcripts identified in pair-wise comparisons and the number of the transcripts upregualted by stage

**Life -stage**	**Total transcripts identified**	**Transcripts**	**Transcripts**
**comparison**	**(adj*P*<0.05)**	**upregulated in**	**upregulated in**
Mf/L3	3,449	Mf-1,370	L3-2,079
L3/L4	2,590	L3-1,197	L4-1,393
L4/6WF	1,979	L4-1,065	6WF-914
L4/6WM	2,910	L4-1,330	6WM-1,580
6WF/F	3,047	6WF-1,878	F-1,169
6WM/M	1,495	6WM-604	M-891
6WF/6WM	2,701	6WF-1,197	6WM-1,504
AM/AF	3,355	AM-2,017	AF-1,338

Mf, microfilariae; AF, adult females; AM, adult males; 6WF, 6 week females; 6WM, 6 week males; L4, L4 stage, 2 weeks post infection; L3, third stage infective larvae.

### *Comparison of genes expressed in Mf and L3*

A comparison of expressed transcripts of Mf and L3 stages identified more differentially expressed transcripts (3,449) than any of the other pairwise comparisons; 2,079 and 1,370 transcripts were upregulated in L3 and Mf, respectively. Distinct expression patterns of the cathepsin L-like cysteine proteinase family were identified in Mf and L3 stages. Transcripts that encode group Ia cathepsin L- like protease enzymes BmCPL-1 (Bm1_00065, Bm1_00870 and Bm1_53625), BmCPL-4 (Bm1_20385, Bm1_00095, and Bm1_13265) and BmCPL-8 (BMC04302) were upregulated in L3, whereas transcripts encoding the group Ic cathepsin L-like protease enzymes BmCPL-2 (Bm1_06180), BmCPL-3 (Bm1_06185) and BmCPL-6 (Bm1_06175 and Bm1_56245) were upregulated in Mf. The group Ia cathepsin L- like proteases were found to be highly expressed in filarial L3 and associated with larval molting and remodeling of the cuticle [26]. Moreover, a recent study shows that *B. malayi* parasites without *Bm-cpl-1* are incapable of completing development to the L3 stage in mosquitoes [44]. However, the putative functions of the group Ic cathepsin L- like protease enzymes are still poorly understood. Upregulation of transcripts for these enzymes in Mf may provide a new clue for understanding the function of these enzymes in filarial parasites.

Enriched transcripts in the pairwise comparison are associated with key biological processes of stage-specific development, such as transition of Mf and L3 to new host. For example, transcripts (BmCHT1- Bm1_28620, Bm1_06755) which encode chitinase necessary for exsheathment of microfilariae [45,46] were upregulated in Mf, and L3s had higher transcription of genes that encode various types of collagen which are important components of L3 cuticle that may be important for protecting the parasites in the mammalian host environments. In addition, L3 expressed transcripts encode proteins involved in pathogenesis, parasitism, and stress resistance that facilitate establishment of infection in a mammalian host. Examples include *B. malayi* abundant larval protein family members BmALT-1 and 2 (Bm1_26880 and BMC12455) that may be used by invading larvae to modulate or evade host immune responses [47] and L3 transcripts (Bm1_14035 and Bm1_14040) that encode a venom allergen-like protein (BmVAL-1) associated with the transition to parasitism and highly expressed in non-activated hookworm L3 [48,49].

We investigated whether L3 upregulated transcripts are enriched potential homologues of *C. elegans* dauer-regulated genes identified by Wang and Kim [50]. BLAST searches identified 64 *B. malayi* L3 upregulated transcripts that are potential homologues of *C. elegans* dauer-regulated genes, and 11 of these are further characterized as dauer-enriched genes (Additional file 8). The dauer-enriched genes presumably may be related to dauer-specific properties such as stress resistance and longevity [50]. Shared expression of homologous genes by *C. elegans* dauer larvae and filarial L3 may provide clues to mechanisms responsible for developmental arrest of filarial L3 in the arthropod host.

In addition to providing corroborating evidence for known-stage-associated genes as stated above, our results identified many transcripts for novel proteins that have not previously been associated with Mf or L3. Information on the timing of expression of novel genes may lead to hypotheses regarding their functions that can be tested with functional genomics.

### *Genes crucial for molting and growth are upregulated in L4 relative to the L3*

The comparison of gene expression between L3 and L4 stages identified 2,590 differentially expressed genes. Of these, 1,393 transcripts were upregulated in L4. Transcripts important for development including stress or heat inducible molecules such as heat shock proteins and anti-oxidants were highly expressed in L4 stage relative to the L3 stage. This pairwise comparison also suggested that protein synthesis is more active in L4s than in L3s, because transcripts encoding many ribosomal proteins (97 different probes) were enriched in L4 compared to the developmentally arrested vector L3 stage. This may be because developing L4 parasites require a high level of biosynthesis to make components for new cuticle as reported in *C. elegans*[51,52]. Genes encoding various collagens and structural proteins such as actin, intermediate filament, and tubulin were also more highly expressed in L4. One of the upregulated collagen genes was Bm1_56360, an ortholog of *C. elegans* gene *bli-1*[5] which is exclusively expressed in L4 in that species and plays a crucial role in molting [52]. In addition, gene Bm1_55540 (an ortholog of *C. elegans* gene *acn-1*, which may regulate the production of peptide molting hormones [52,53]), was also highly expressed in *B. malayi* L4. Thus, transcription profiles appear to illustrate how filarial parasites coordinate gene expression with biological processes such as molting and growth in the mammalian host.

#### Pairwise comparisons of sexually immature and mature worms revealed temporal differences in gender-associated gene expression

Prior studies showed that 6WM and 6WF *B. malayi* worms (recovered from peritoneal cavity of jird) are not sexually mature and are still growing [53,54]. Transcripts differentially expressed in pairwise comparisons between sequential stages (L4 vs. 6WF, L4 vs. 6WM, 6WF vs.AF and 6WM vs. AM) are likely to be important for sexual development. Results of these comparisons were consistent with this hypothesis and with the findings of prior studies of gender-associated genes [6,8]. For example, transcripts for female-associated proteins such as high mobility binding protein and caveolin (Bm1_25620 and Bm1_36280) were enriched in growing 6WF parasites and transcripts for male-associated proteins such as major sperm protein and PDZ domain protein (Bm1_55755 and Bm1_14680) were abundant in growing 6WM parasites relative to L4 larvae. Likewise, transcripts for cathepsin protein gene family members BmCPL (Cathepsin L), BmCPF (cathepsin F), BmCPZ (cathepsin Z), and BmCPQ (cathepsin Q) that are essential for early sexual development and early embryogenesis in *B. malayi* parasites [26,55-57] were highly expressed in both 6WF and 6WM relative to L4 and adult worms (AM and AF).

Interestingly, some of the transcriptomic changes during sexual development were quite different in females and males. Many more upregulated transcripts (1,580) were found in 6WM stage than in 6WF (914) relative to L4. These results are consistent with known temporal differences in gender development of *B. malayi*. Prior studies have shown that sexual development occurs earlier in males than in females in *B. pahangi*[58,59], and spermatogenesis occurs prior to oogenesis in *C. elegans* hermaphrodites [60,61].

**Figure 4:**
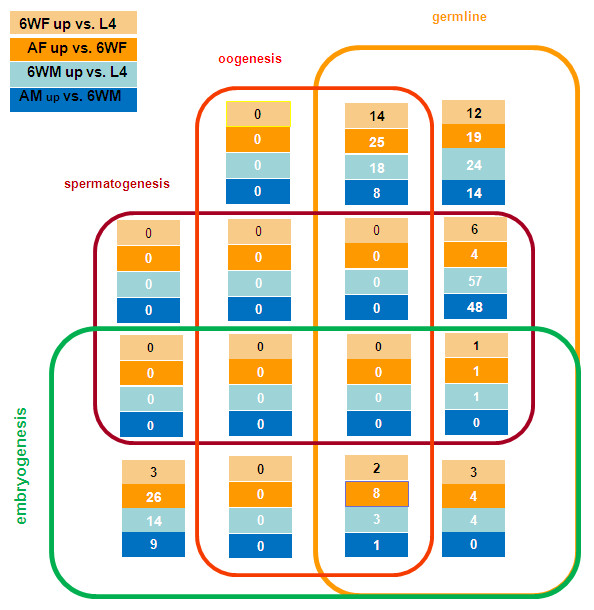
**Distribution of the potential*C. elegans*homologues identified in gender-associated transcripts in pair-wise comparisons**. These homologues were annotated as “germline-, oogenesis-, spermatogenesis-, and embryogenesis-enriched”. The numbers in each box represent the number of potential filarial homologues in each category.

It has been proposed that the *C. elegans* genome can serve as a guide to examine aspects of the biology of parasitic nematode species [62], and we have shown that this comparative genomics approach has great power. When we cross-referenced upregulated transcripts in 6WF and 6WM relative to L4 with global expression patterns in *C. elegans* reported by Reinke et al. [32], 121 potential *C. elegans* homologues that are germline-, spermatogenesis-, oogenesis-, or embryogenesis-enriched were identified in 6WM upregulated transcripts. In contrast, only 41 were identified in 6WF upregulated transcripts relative to L4 (Figure 4 and Additional file 9). Again these results are consistent with the earlier sexual development of male filarial worms compared to females.

### *Comparison of gene sets associated with development identified by clustering and by pair-wise comparison of gene expression*

As expected, there was a significant overlap between the gene sets identified by these two approaches. For example, 978 of 1,038 of Mf enriched transcripts in Cluster one (94%) and 777 of 882 L3 enriched transcripts in Cluster two (88%) identified by hierarchical clustering were consistent with results obtained by pairwise comparisons, respectively. We have especially high confidence in transcription differences that were identified by both clustering and pairwise comparisons. Of course, pairwise analyses tend to identify more stage-regulated genes than global expression analysis. For example, L4 enriched transcripts identified in the L3 vs. L4 pairwise comparison (N = 1,393) far outnumbered the number of transcripts in Cluster three (L4 enriched, N = 786).

### *Gene ontology associations for developmentally regulated genes*

A file containing all GO terms across all three categories (Biological_Process, Molecular_ Function or Cellular_Composition) mapped to V2 probes was generated and posted at Nematode.net [63] (http://www.nematode.net/NN3_frontpage.cgi?navbar_selection=funcexp&subnav_selection=microarray). The full lists of enriched GO terms are provided in supplemental datasets. The significantly enriched GO associations annotated in various gene sets are present in additional files, genes constitutively expressed (Additional file 10), genes expressed in Five clusters (Additional file 11) and genes differentially expressed in pairwise comparisons (Additional file 12).

GO enriched Molecular Function categories for upregulated transcripts in AM and AF were consistent with previously reported GO categories for gender enriched transcripts [8]. For example, AM have enriched transcripts for protein tyrosine phosphatase activity (GO:0004725) and protein kinase activity (GO:0004672) whereas AF contain enriched transcripts associated with protein synthesis such as peptidyl-prolyl cis-trans isomerase activity (GO:0003755) and structural constituent of ribosome (GO:0003735).

Enriched GO terms in both Molecular Function and Biological Process tended to be quite distinct in different gene sets. For example, the majority of constitutively expressed gene products have GO term annotations that place their putative functional role in fundamentally important ‘house-keeping’ processes such as translation (GO:0006412) and response to heat and stress (GO:0009408 and GO:0006950) ( Additional file 10). The significantly enriched functional classes varied quite a bit between Clusters 1–5 ( Additional file 11), and they were consistent with the main biological processes for corresponding life cycle stages. The prominent biological processes in Cluster 3 (L4 enriched) were obviously related to events in the parasites’ development and survival in the mammalian host. These included immune response (GO:0006955) and responses to stress (GO:0006950) for survival and glycolysis (GO:0006096) for energy supply. Coordinately, enriched molecular function classes related to biological processes in Cluster 3 included antioxidant activity (GO:0016209) and phosphogluconate dehydrogenase (decarboxylating) activity (GO:0004616). By contrast, chitin catabolic process (GO:0006032) was one of the most significantly enriched biological processes in Cluster one (Mf stage enriched), and chitinase activity (GO:0004568) was a significantly enriched molecular function assigned to transcripts in that cluster.

**Figure 5:**
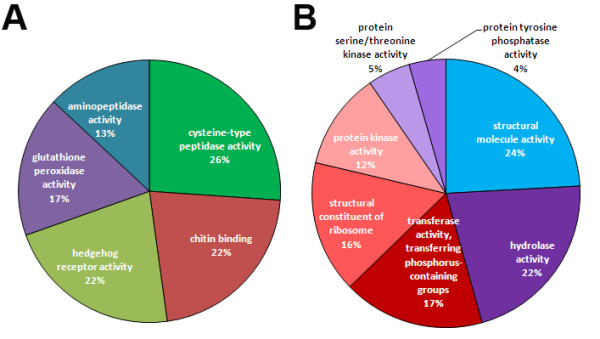
**Infections are initiated after Functional categories of transcripts upregulated in*B. malayi*young adult worms identified by pair-wise comparison**. Pie charts show functional annotations for each of the major gene sets based on gene ontology (GO) annotation. **A**: Functional categories for 6WF upregulated transcripts vs. L4; **B**: Functional categories of 6WM upregulated transcripts vs. L4. The molecular functions were quite distinct between these two gene sets.

While enriched GO annotations for clusters provide a broad view of the potential functions of molecules with stage-dependent expression patterns, GO enrichments assigned to upregulated transcripts in the various pair-wise comparisons provide further insight into molecular functions and biological processes that are associated with stage-specific transitions ( Additional file 12). For example, differentially expressed transcripts in 6WF relative to L4 have enriched GO terms with putative functional roles in the biological processes of response to stress, heat, oxidative stress and proteolysis, whereas the differentially expressed transcripts in 6WM over L4 have enriched GO terms with potential molecular functions of protein kinase, phosphatase and transferase activity, and ribosome structure (Figure 5 A and B). These results suggest that 6 week (immature adult) worms already have sex-specific transcription patterns associated with gender-associated biological processes.

### *Comparison of B. malayi transcriptomic data with published proteomic data*

The integration of transcriptomic and proteomic data is a huge challenge. Until now, only a few published reports have attempted to do this on a large scale, and the results typically reveal only modest correlations between those two types of data [64-67]. With this in mind, we manually compared our transcriptome data with recently published *B. malayi* proteome data [10-13]. Among the proteins from five lifecycle stages (adult male, adult female, blood-borne and uterine microfilariae and infective L3 larvae) identified by Bennuru et al. [13], 63% (3,675 out of the 5,852 proteins that correspond to probes on array) had expression signals detected in the current study (Additional file 13). Obviously, the proteome study did not report proteins for many transcripts detected in the present study. These differences are not surprising given limitations of the methods and differences in timing between transcription, translation, and detection of proteins in worms.

When we compared current transcriptional and proteomic profiles described by Bennuru et al. [13] in more detail, one of the striking findings was that the majority of transcripts with proteomic data (69%, 2,552 out of 3675 genes) were not differentially expressed across the lifecycle. This indicates that the proteins differentially expressed are less abundant in proteomic data. Among the 31% of proteins that were differentially expressed (1,122 out of 3,675), the highest percentage (34%) was observed in Cluster five (male-enriched). Some discrepancies between stage-related transcriptional and translational profiles may be biologically relevant. For example, the major microfilarial sheath protein (BmSHP) encoded by gene *Bm-shp-1* (Bm1_19100) was enriched in the microfilarial proteome [13], while the transcript for BmSHP (Bm1_19100) was enriched in the female –enriched cluster (Cluster four) and not in the microfilarial cluster (Cluster one). These results are consistent with results from a prior *in situ s*tudy that showed that *Bm-shp-1*was exclusively expressed in the uterine epithelium of female worms with no expression signal in embryos or microfilariae [68]. This type of information may be critically important for selection of candidate genes for RNAi experiments.

## Conclusion

This study has provided detailed information on changes in gene expression across lifecycle stages of the filarial parasite *B. malayi*. Results in the current study are consistent with findings previously reported from transcriptome studies that focused on L3 and sexually mature adult male and adult female worms[7,8]. Approximately 61% of gender-associated transcripts from our previous study [8] were confirmed in the pairwise comparison of AF vs. AM in the current study. This consistency supports the validity of the current data, which show that gene expression is highly regulated and finely tuned across the *B. malayi* life cycle. Some genes were expressed in all stages at fairly constant levels, while expression of other genes varied by 10 to 80-fold. Many of these changes were consistent with parasite biology. For example, the transition of the parasite from the mosquito vector to the mammalian host was associated with increased transcription of genes involved in invasion, immune interactions, and molting, while the transition from immature to sexually mature stages disproportionately affect transcription of genes required for reproduction.

This study has provided a detailed description of transcriptional events across the life cycle of *B. malayi*. We have illustrated how global gene expression profiles can provide vital information on the development and biology of an important filarial parasite. Transcriptome information is complementary to that produced by genome sequencing and proteomics. By identifying stage-specific transcripts and other genes that are regulated across the life cycle, this study has shed new light on mechanisms used by these highly evolved parasites to establish infections and to survive and reproduce in their hosts.

## Methods

### *Ethics statement*

The animal work was carried out under protocols #20050377 and 20090045 that were approved by the Animal Studies Committee of Washington University School of Medicine at St Louis, Missouri, USA.

### *Collection of B. malayi* life cycle stages

The life cycle of *B. malayi* and stages studied are shown in ( Additional file 1: Figure S1). Different stages at 2 wk post infection (L4), 6 and 12 week post infection of *B. malayi* worms (TRS strain) were isolated from the peritoneal cavity of i.p. infected jirds (*Meriones unguiculatus)* obtained from the NIAID Filariasis Research Reagent Resource Center (FR3) (University of Georgia, Athens, GA) as previously described [7]. Male and female worms collected from the animals 6 and 12 week post infection were carefully sexed by size and morphology. The worms were carefully washed in PBS and immediately frozen at –80^o^ C. Microfilariae directly from the peritoneal cavity of infected jirds, and L3 larvae from mosquitoes were directly obtained from FR3.

### *RNA isolation, probe preparation and microarray hybridization*

Total RNA was prepared from 30 mature adult worms, 1,000 L4, 1,000 6 week female, 1,000 6 week male, 3,000 L3, approximately 10,000 microfilariae per batch using TRIzol (GibcoBRL, Life Technologies) as previously described [6]. cDNA was synthesized from 5–7 ug female or male total RNA using 3DNA capture sequence primers (3DNA Array 350 Detection system, Genisphere, Hatfield, PA) and SuperScript II Reverse Transcriptase (Gibco BRL, Gaithersburg, MD) for each probe according to standard protocols. cDNA was concentrated using a Microcon YM-100 filter (Millipore) and either used immediately or stored at −80 ^0^ C. cDNA synthesized from two different batches of each RNA sample that was independently prepared were used as biological replicates. A two-step protocol was used for hybridization (3DNA Array 350 Detection system, Genisphere, Hatfield, PA) as previously described [6]. Each experiment consisted of a pair-wise competitive hybridization of cDNA samples (stage/common reference pool) with reciprocal dye-flip replicates. A common mRNA reference pool was made from pooled mRNA with an equal amount of mRNA from each stage and representing all stages of life cycle studied. Because biological replicates and dye-flip replicates were tested, a total of four DNA microarrays were used for each comparison of two types of cDNA. Eight hybridizations were performed for each element on the array, as all probes are present in duplicate on the array.

### *Microarray fabrication*

The BmV2 array contains 18,104 elements derived from *B. malayi* (15,412), *Onchocerca volvulus* (1,016), *Wuchereria bancrofti* (872) and *Wolbachi*a (*wBm*, 804 genomic elements. All information regarding the BmV2 array including oligo name, sequence and source corresponding to the *B. malayi* genome (Bm1_nomenclature) and *B. malayi* peptide models (XXXXX.m00YY nomenclature) and *B. malayi* gene index (ESTs, TC nomenclature) and full length consensus sequences are available from http://www.filariasiscenter.org/brugia-malayi-genomics-and-bioinformatics-resources/. The features of the BmV2 array have been previously described [7]. The oligonucleotides (50 nM in 3x SSC with 0.75 M betaine) were printed in duplicate on MWG Epoxy slides (MWG Bioteche Inc, High Point, NC) by a locally constructed linear servo-arrayer (after the DeRisi model, http://h ).

### *Microarray data extraction*

Arrays slides were scanned immediately after hybridization on a ScanArray Express HT Scanner (Perkin Elmer,Boston, MA) to detect Cy3 and Cy5 fluorescence at 543 and 633 nm, respectively, and were analyzed with Bioconductor software [69]. After scanning, background correction was done to reduce the effect of background noise, followed by normalization with the Lowess method, and normalized array data were fitted into linear models to detect differential expression over different stages. These procedures were carried out with packages from Bioconductor, among them the linear model fitting was carried out with LIMMA of Bioconductor [70]. Normalized microarray data were also examined to determining gene expression presence. A gene was deemed “Present” in a specific stage when its normalized intensities of three out of the four replications of that stages not less than two folds of the mean intensity of negative controls. Differential expressed genes were defined as those with 1) multiple corrected P < 0.05 (F test) in the above mentioned linear model fitting with LIMMA and 2) at least “Present” in one stage. Pair-wise comparison was further carried out for all differentially expressed genes between interested stages. A total seven pair-wise comparisons were carried out. Significance was defined as P < 0.05 (*T* test). These were also done with LIMMA. All microarray data are MIAMI compliant and a full (including controls) set of raw and normalized data is available via Nematode.net [63] and raw data can be downloaded through http://www.nematode.net/NN3_frontpage.cgi?navbar_selection=funcexp&subnav_selection=microarray.

### *Identification of gene sets associated with development*

The differentially expressed genes (transcripts) detected using LIMMA were clustered based on their expression profile over different stages using K-means clustering implemented in Clara (http://astrostatistics.psu.edu/su07/R/html/cluster/html/clara.html). The optimal number of clusters (5 in this study) was decided by comparing the silhouette information of clustering with different clusters.

### *Gene ontology (GO) analysis*

Gene ontology annotation was done using InterProScan taking the default cutoff. GO term enrichment/depletion was detected using FUNC [71]. Multiple test corrected P < 0.05 was used to define enrichment/depletion. A hypergeometric test was performed to identify GO terms that were enriched in various gene sets, and GO terms having a hypergeometric P-value < 0.05 were considered to be enriched.

### *Identification of potential B. malayi homologues of C. elegans genes annotated as “embryogenesis-, spermatogenesis-, oogenesis and germline-enriched”, and “dauer-enriched”*

The full length consensus sequences corresponding to BmV2 18,104 probe sets were queried against the non-redundant (NR) protein database of the National Center for Biotechnology Information (NCBI) and a *C. elegans* database (Wormpep 195) as previously described [8]. The best potential homologues were reported with a probability of 1e-05 or better. The best potential *B. malayi* homologues of *C. elegans* were identified in each expression data set. We then searched in-house stored *C. elegans* data sets including 1,984 -dauer regulated and 540 dauer-enriched genes [50] and germline- and sex-regulated genes [32] and the potential filarial homologues of *C. elegans* genes in those data sets were reported in each expression data set.

## Abbreviation

LF, lymphatic filariasis; L3, infective larvae; Mf, microfilariae; 6WF, female worm collected 6 week post infected; 6WM, male worm collected 6 week post infected; AF, mature adult female worm collected 12 week post infected; AM, mature adult male worm collected 12 week post infected.

## Competing interests

The authors declare that they have no competing interests.

## Authors’ contributions

BWL designed experiment, data mining, and analysis and drafted the manuscripts; ZW performed bioinformatical and statistical analyses; AR participated in data mining, analysis and qRT-PCR; MM participated in data analysis; GW designed experiment and drafted manuscript. All authors read and approved the final manuscript.

## Supplementary Material

Additional file 1 **The schema of*B. malayi*lifecycle and stages studied in current study.** Figure S1. This figure shows the life cycle stages of *Brugia malayi* and lifecycle stages included in current study. The life cycle of the filarial parasite is digenetic with a mammalian host (light green) and a mosquito intermediate host (green). Infections are initiated after a mosquito blood meal when the infective L3 stage enter the mammalian host. L3 larvae migrate to lymphatic vessels, develop, and molt twice to transform into adult parasites. The adult parasites mate, produce microfilariae (Mf) that are released into the circulation and ingested by mosquitoes during a blood meal. The ingested microfilariae molt twice and develop into the infective L3 stage in mosquito vector. Note: Stages analyzed in this study are marked in black circles. Click here for file

Additional file 2 Transcripts expressed in any stage and gender. Click here for file

Additional file 3 **Differentially expressed transcripts (DE) identified across the stages studied (adj*P* < 0.05).** Click here for file

Additional file 4 Five transcriptional patterns identified by K-means clustering. Click here for file

Additional file 5 **Potential filarial homologues of*C. elegans*genes annotated as “spermatogenesis- and oogenesis-enriched” identified in the clusters.** Click here for file

Additional file 6 Transcriptional profiles of gene family identified in global transcription data. Click here for file

Additional file 7 Differentially expressed transcripts identified in pairwise comparisons. Click here for file

Additional file 8 **Potential filarial homologues of*C. elegans*genes annotated as “dauer-regulated and dauer-enriched” identified in L3 upregulated transcripts vs. Mf.** Click here for file

Additional file 9 **Potential filarial homologues of*C. elegans*genes annotated as spermatogenesis-, oogenesis-, embryogenesis- and germline-enriched identified within data sets: upregulated transcripts in 6WM vs. L4, upregulated transcripts in 6WF vs. L4, upregulated transcripts in AM vs. 6WM and upregulated transcripts in AF vs. 6WF.** Click here for file

Additional file 10 GO associations of transcripts constitutively expressed across the stages studied. Click here for file

Additional file 11 GO associations of transcripts expressed in five gene clusters. Click here for file

Additional file 12 GO associations of transcripts differentially expressed in pairwise comparisons. Click here for file

Additional file 13 Expressed transcripts with encoded proteins identified by previous proteomic studies. Click here for file
