# Supplementary figures and images for: Transcription profiling reveals stage- and function-dependent expression patterns in the filarial nematode Brugia malayi
Source: BMC Genomics. 2012 May 14;13:184. doi: 10.1186/1471-2164-13-184 (PMC3414817; doi:10.1186/1471-2164-13-184)

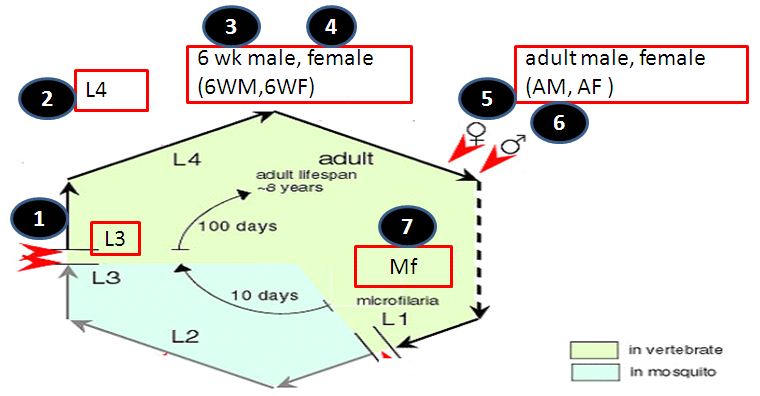

Supplement: Additional file 1 — The schema ofB. malayilifecycle and stages studied in current study. Figure S1. This figure shows the life cycle stages of Brugia malayi and lifecycle stages included in current study. The life cycle of the filarial parasite is digenetic with a mammalian host (light green) and a mosquito intermediate host (green). Infections are initiated after a mosquito blood meal when the infective L3 stage enter the mammalian host. L3 larvae migrate to lymphatic vessels, develop, and molt twice to transform into adult parasites. The adult parasites mate, produce microfilariae (Mf) that are released into the circulation and ingested by mosquitoes during a blood meal. The ingested microfilariae molt twice and develop into the infective L3 stage in mosquito vector. Note: Stages analyzed in this study are marked in black circles. [file 1471-2164-13-184-S1.tiff]
